# Erichsen on the Elastic Ligature

**Published:** 1874-07

**Authors:** 


Erichsen on the Elastic Ligature.—In a letter to the *Medical Times and Gazette,* Mr. John Erichsen characterizes the use of Prof. Dittel's elastic ligature as "simply a return to "mediæval barbarism."

